# Quality-of-life benefits and evidence of antitumour activity for patients with brain metastases treated with gefitinib

**DOI:** 10.1038/sj.bjc.6601478

**Published:** 2003-12-10

**Authors:** A Katz, P Zalewski

**Affiliations:** 1 Centro Paulista de Oncologia and Department of Oncology, Albert Einstein Hospital, São Paulo, Brazil; 2 The Scarborough Hospital, Scarborough, Ontario, Canada

**Keywords:** gefitinib (‘Iressa’, ZD1839), EGFR, quality of life, brain metastases, antitumour activity

## Abstract

Brain metastases are a common complication of non-small-cell lung cancer (NSCLC). The role of chemotherapy in the treatment of brain metastases has not been clearly defined. Emerging case reports of patients with recurrent NSCLC treated as part of the Expanded Access Programme reveal that gefitinib (‘Iressa’, ZD1839) has clinical activity in some patients with brain metastases. Here, we describe a number of case studies documenting the response of patients with brain metastases to treatment with gefitinib. Many of these patients had quality-of-life benefits with improvement of neurological and systemic symptoms; some had a partial response of their brain metastases and even complete responses have been seen in a few patients. One case report also describes a durable long-term response with concurrent treatment with gefitinib and radiotherapy. Such results call for larger trials designed to evaluate and define the role of gefitinib in the treatment of brain metastases in NSCLC patients, either as a single agent or in combination with radiation therapy.

Brain metastases are a common complication for patients with non-small-cell lung cancer (NSCLC) and a significant cause of morbidity and mortality. Radiotherapy remains the mainstay of treatment for brain metastases; however, patients treated with whole-brain radiation still have a poor prognosis with a median survival of only 3–4 months (Rodrigus *et al*, 2001). The role of chemotherapy in the treatment of patients with brain metastases has not been clearly defined. Many of these tumours are relatively resistant to chemotherapy and, traditionally, it has been assumed that the blood–brain barrier prevented chemotherapeutic agents from entering the central nervous system (CNS). However, the blood–brain barrier is disrupted in malignant brain tumours and recent evidence suggests that the chemosensitivity of the primary tumour is the major determinant of the response to systemic treatment of brain metastases, rather than the ability of the agent to penetrate an intact blood–brain barrier.

Gefitinib (‘Iressa’, ZD1839) is an orally active, epidermal growth factor receptor (EGFR) tyrosine kinase inhibitor that blocks signal transduction pathways implicated in the proliferation and survival of cancer cells. High levels of EGFR are frequently found in malignant primary brain tumours (Ekstrand *et al*, 1992) and in tumours that often metastasise to the CNS, such as NSCLC and breast cancer (Wikstrand *et al*, 1995). Large Phase II studies have recently shown that gefitinib has clinical activity in advanced NSCLC and is well tolerated (Fukuoka *et al*, 2003; Kris *et al*, 2003). A preclinical study in mice showed that gefitinib retains therapeutic activity on intracranial tumours despite the blood–brain barrier (Heimberger *et al*, 2002). Oral administration of gefitinib at 50 or 100 mg kg^−1^ day^−1^ for 3 weeks in athymic mice with established intracerebral A431 human epidermoid carcinoma expressing EGFR increased median survival by 88% (*P*=0.009) and 105% (*P*<0.001), respectively. The preliminary results of a Phase II study also show modest activity of gefitinib in patients with recurrent glioblastoma (Peery *et al*, 2003).

Recently, a number of case reports of patients with recurrent NSCLC treated with gefitinib as part of the Expanded Access Programme (EAP) have described promising activity in patients with brain metastases. Here, we accumulate these results and discuss the possible implications and future directions.

## GEFITINIB IN PATIENTS WITH BRAIN METASTASES: RESULTS FROM THE EAP

The case reports of patients with brain metastases secondary to NSCLC treated with gefitinib as part of the compassionate-use programme have recently been reported as published case studies (Cappuzzo *et al*, 2003a, 2003b; Fujiwara *et al*, 2003; Knuti *et al*, 2003; Villano *et al*, 2003), at the World Conference on Lung Cancer 2003 (Chiu *et al*, 2003; Pino *et al*, 2003), at the American Society of Clinical Oncology 2003 conference (Ceresoli *et al*, 2003) and at the ‘Iressa’ Clinical Experience (ICE) meeting (Awada, ICE abs; Cappuzzo [a & b], ICE abs; Dieriks, ICE abs; Stein, ICE abs; de la Cruz [a & b], ICE abs; Maione, ICE abs; Martínez, ICE abs; Diaz-Cantón, ICE abs; van der Kamp, ICE abs; van Zandwijk [b], ICE abs; Petruzelka [a], ICE abs; Azémar, ICE abs; Roggero, ICE abs; Kowalski, ICE abs; Martín-Algarra, ICE abs; Pavlakis, ICE abs). (See appendix for ICE abstracts). Together, these case reports suggest that gefitinib has activity in NSCLC brain metastases. The results of published case series are shown in Table 1

**Table 1:** Published case series of patients with brain metastases and non-small-cell lung cancer treated with gefitinib as part of the Expanded Access Programme

	**Patients**	**Previous treatment**	**Results**
Pino *et al* (2003)	*n*=63, of whom 11 patients	One prior chemotherapy regimen: 18	Of 11 patients with brain metastases,
	had brain metastases	Two prior chemotherapy regimens: 42	two had a response in the brain
Chiu *et al* (2003)	*n*=57, of whom 16 had brain metastases	One prior chemotherapy regimen: 1	Of 16 patients with brain metastases,
		⩾2 prior chemotherapy regimens: 15	two had a partial response
Ceresoli *et al* (2003)	*n*=27, all with brain metastases	Platinum-based chemotherapy: 20	Two patients had a response in the brain,
		Brain radiotherapy: 11	one with complete remission (both had brain radiotherapy >3 months before starting gefitinib)
Cappuzzo *et al* (2003a)	*n*=4, all with brain metastases	⩾2 prior chemotherapy regimens: 4Whole-brain radiotherapy: 3	One patient had a complete response and three patients had a partial response of brain metastases
			Response duration: 3+, 5, 7, 12+ months
Cappuzzo *et al* (2003b)	*n*=4, all with brain metastases	One prior chemotherapy regimen: 2⩾2 prior chemotherapy regimens: 2	All four patients had a partial response of brain metastases
		Whole-brain radiotherapy: 2	Response duration: 6+, 8, 11+, 15 months

.

**Figure 1:**
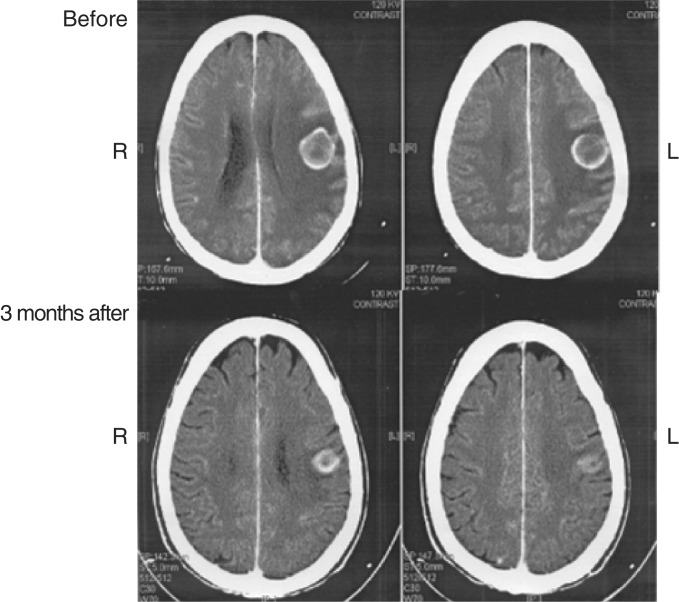
Computed tomography scan before and after treatment with gefitinib. Reproduced with permission from: Cappuzzo F, Calandri C, Bartolini S, Crino L (2003b).

Most notable are the cases with a complete remission of brain metastases. In the largest case series that included 27 patients who had asymptomatic or symptomatic brain metastases, two of 20 evaluable patients had a response in the brain, with complete remission in one patient (Ceresoli *et al*, 2003). In another case series described in two separate publications by Cappuzzo *et al*, eight patients with NSCLC and brain metastases were treated with gefitinib (Cappuzzo *et al*, 2003a, 2003b; [a & b] ICE abs). One patient had a complete response and seven had partial responses in the brain, all within 3 months of treatment. The complete response in the brain was observed 6 weeks after treatment with gefitinib started in a patient who had completed whole-brain radiotherapy 3 months prior to the beginning of gefitinib (Figure 1

). All eight patients experienced improvements in neurological and systemic symptoms.

Fujiwara *et al* (2003) described the case report of a patient who had achieved a complete response in the brain following treatment with gefitinib. This particular patient was diagnosed in July 1999 with stage IV adenocarcinoma of the left lung. In March 2001, after treatment with three different chemotherapy regimens, asymptomatic brain metastases were detected and subsequently treated with whole-brain radiotherapy. However, by October 2001 the patient's condition had deteriorated, with progressive brain metastases, and the patient was readmitted to hospital. Treatment with gefitinib started in November 2001 after which computed tomography scans showed that brain metastases had disappeared and that there was a marked regression of the lung tumour. The patient was then discharged in December 2001 and was able to resume full-time work for nearly 8 months. Two other published case reports also describe patients whose brain metastases responded to treatment (Knuti *et al*, 2003; Villano *et al*, 2003). In all three of these cases, patients had worsening neurological symptoms and deteriorating performance status before treatment with gefitinib. Following treatment, a significant clinical improvement was seen in all three patients: brain metastases disappeared in the patient already described and the sizes of the other two patients' lesions decreased. Quality of life was improved in all three patients.

**Figure 2:**
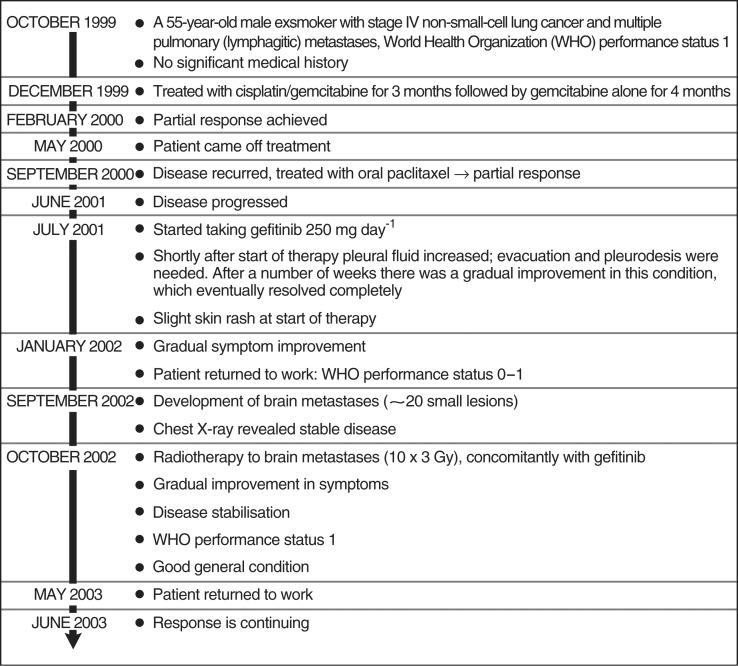
Case history of a patient with NSCLC and brain metastases taking gefitinib. Data previously presented by: van Zandwijk N. Risk/benefit of gefitinib (‘Iressa’, ZD1839). Oral presentation at the ICE meeting, Madrid, June 2003. Used with permission.

**Figure 3:**
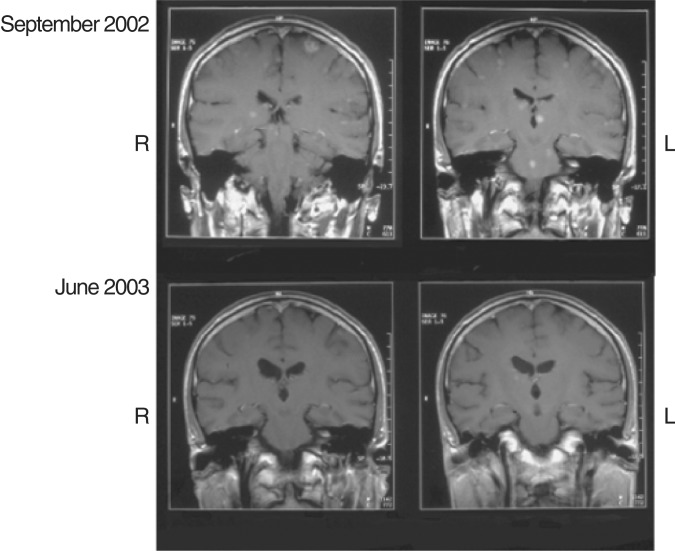
Computed tomography scan before and after treatment with gefitinib and radiotherapy (case history shown in Figure 2). Previously presented by: van Zandwijk N. Risk/benefit of gefitinib (‘Iressa’, ZD1839). Oral presentation at the ICE meeting, Madrid, June 2003. Reproduced with permission.

One patient described at the ICE meeting showed good tolerance of concurrent gefitinib and radiotherapy (van Zandwijk [b], ICE abs, personal communication). In this case study, a 55-year-old male exsmoker with stage IV NSCLC and multiple pulmonary (lymphangitic) metastases developed multiple brain metastases (approximately 20 small lesions) after receiving treatment with gefitinib for >1 year (Figure 2

). Radiotherapy to the brain (10 × 3 Gy) and continued treatment with gefitinib resulted in disease stabilisation and symptom improvement and the patient was able to return to work. Delayed imaging 9 months later showed almost complete resolution of the brain metastases (Figure 3

). Thus, although gefitinib did not prevent the development of brain metastases, concurrent treatment with radiotherapy and gefitinib resulted in a durable clinical response.

In total, 25 case reports of gefitinib use in patients with brain metastases and NSCLC were described at the ICE meeting (including the eight patients described by Cappuzzo *et al*) (Awada, ICE abs; Cappuzzo [a & b], ICE abs; Dieriks, ICE abs; Stein, ICE abs; de la Cruz [a & b], ICE abs; Maione, ICE abs; Martínez, ICE abs; Diaz-Cantón, ICE abs; van der Kamp, ICE abs; van Zandwijk [b], ICE abs; Petruzelka [a], ICE abs; Azémar, ICE abs; Roggero, ICE abs; Kowalski, ICE abs; Martín-Algarra, ICE abs; Pavlakis, ICE abs). Of these, three patients had a complete response, 10 a partial response, eight had stable disease and four patients had progression of their brain metastases. In all of these case reports, treatment with gefitinib appears to have been well tolerated. Any reported adverse events are consistent with the adverse-event profile of gefitinib established in Phase II trials in patients with advanced NSCLC (Fukuoka *et al*, 2003; Kris *et al*, 2003). There was also no unexpected or cumulative toxicity described for the patient who received concurrent gefitinib and radiotherapy (van Zandwijk [b], ICE abs).

## DISCUSSION

A number of case reports suggest that gefitinib has clinical activity against brain metastases of patients with NSCLC. These preliminary data indicate that patients with brain metastases should not be excluded from treatment with gefitinib (as they were in some of the early clinical trials of gefitinib), an opinion also expressed by Cappuzzo *et al* (2003b). There is a need for larger trials to follow-up these positive preliminary findings in the hope of developing a treatment option for these patients who have a very poor prognosis. Combination of radiotherapy and gefitinib administered either concurrently or sequentially warrants further investigation.

A consideration for such future studies in patients with brain metastases is the optimum dose of gefitinib if dexamethasone is also being administered. Dexamethasone is an inducer of cytochrome *P*450 3A isoenzymes, including CYP 3A4, which has been identified as the isoenzyme primarily involved in the metabolism of gefitinib. Therefore, coadministration of dexamethasone may lead to increased metabolism of gefitinib, with consequently lower plasma concentrations and potentially reduced efficacy. In addition, such studies would need to use standardised patient staging to identify and monitor asymptomatic as well as symptomatic brain metastases.

A number of preclinical studies support the combined use of radiotherapy and gefitinib as a strategy for cancer treatment (Bianco *et al*, 2002; Huang *et al*, 2002; Magne *et al*, 2002; Williams *et al*, 2002; Solomon *et al*, 2003). Coadministration of gefitinib potentiated the antitumour effect of radiation on A431 cells (human vulvar squamous-cell carcinoma cells that express high levels of EGFR) both *in vitro* and *in vivo* (Solomon *et al*, 2003). Another preclinical study showed a cooperative antiproliferative effect when gefitinib was combined with radiation in colon, ovarian, NSCLC and breast cancer cell lines (Bianco *et al*, 2002). Further, the combination of gefitinib and radiation resulted in long-term tumour growth regressions of established human colon cancer GEO and lung adenocarcinoma A549 xenografts (Bianco *et al*, 2002). Radiotherapy treatment may also disrupt the blood–brain barrier (van Vulpen *et al*, 2002) that would allow improved access of gefitinib to brain tumour tissue.

In summary, a number of case reports suggest activity of gefitinib against brain metastases secondary to advanced NSCLC. Larger trials are needed to validate these findings and further investigate the potential of combined gefitinib and radiotherapy treatment.
